# Extended liver resection in mice: state of the art and pitfalls—a systematic review

**DOI:** 10.1186/s40001-020-00478-3

**Published:** 2021-01-09

**Authors:** Can Kamali, Kaan Kamali, Philipp Brunnbauer, Katrin Splith, Johann Pratschke, Moritz Schmelzle, Felix Krenzien

**Affiliations:** Department of Surgery, Campus Charité Mitte and Campus Virchow-Klinikum, Charité - Universitätsmedizin Berlin, Augustenburger Platz 1, 13353 Berlin, Germany

**Keywords:** Mice, Liver regeneration, Animal experimentation, Hepatectomy, Liver resection, Mortality, Suture ligation, Hemostatic clip

## Abstract

**Background:**

Rodent models of liver resection have been used to investigate and evaluate the liver’s complex physiology and pathology since 1931. First documented by Higgins and Anderson, such models were created to understand liver regeneration mechanisms to improve outcomes in patients undergoing extensive liver resection for liver cancer or other underlying liver diseases.

**Methods:**

A systematic search was conducted using Pubmed, gathering publications up to January 2019, which engaged with the mouse model of extended liver resection as a method itself. The results of this search were filtered according to their language, novelty, and relevancy.

**Results:**

The Boolean search found 3741 articles on Pubmed, with 3130 publications remaining when filtered by language and the presence of a full text. In total, 21 of these publications examined the key themes of the animal model described. The mortality varied from 0 to 50% depending on the surgeon's experience and the resection method. The liver resection was mainly performed with classic sutures (14 out of 21 publications) and isoflurane was used for anaesthesia (10 out of 21 publications) in combination with analgesics (buprenorphine or ketamine/xylazine). The most used mouse strain was C57BL/6 (7 of 21 publications) which was on average 11 weeks old with a weight of 23 g.

**Conclusion:**

Through the overview, laid out in the selected publications, this paper reviews the shift of the extended liver resection model from rat to the mouse, describes the state of the art in the experimental setting, and discusses the possible limitations and pitfalls. Clearly, the extended liver resection in mice is a reproducible, practical and easy to learn method.

## Background

The liver’s capability to regenerate itself after surgical removal of organ parts up to a specific amount is a well-known phenomenon. Clinicians make use of this capability, especially in oncological cases, where the resection of the tumour can be the treatment of choice when looking for a curative approach [1, 2]. The deciding factor in these patients’ clinical outcome is the remnant liver volume and its rest functionality [3–5]. Depending on the extent [6] of the liver resection, the acute injury of the liver can be intercepted by the organs phenomenal regenerative capacity. Many common factors, including chemotherapy, viral infections, chronic inflammation, and fatty liver disease, contribute to the liver’s pathological healing process and its reduction of normal regenerative capacity with evidence of fibrosis and cirrhosis [7, 8].

Recent improvements in preoperative diagnostics, liver capacity tests (e.g., LiMAx), laparoscopic and robotic techniques, and postoperative care have led to improved postoperative outcomes [9–11]. However, in the case of large tumour load and a possible pre-damaged liver due to the chemotherapy or liver cirrhosis, therapeutic options are limited [12]. An excessively-small remnant volume causes liver insufficiency and liver failure. Therefore, liver regeneration remains crucial to enable liver surgery even in advanced tumour stages or in patients with underlying liver diseases. Prediction of the liver insufficiency, a priori would reduce mortality, morbidity, and the hospitalization time [13, 14].

In order to comprehend the complex biological, biochemical, and physiological mechanisms behind the regenerating liver, in vitro and in vivo experimental models provide different benefits. While the in vitro models offer isolated cell groups, where one can explore the details of signalling pathways, trans-membrane activities, and receptor functions, in vivo models have the advantage of depicting the cross-talk of metabolites and between different cells, tissues, and organs [9].

Animal models provide stable and reproducible experimental designs, which successfully reflect the human analogies and have been used since the pioneering publication by Higgins and Anderson in 1931 [5, 7, 8, 15–17]. Since then, the described rat model has been very well documented, and now serves as the main method by which to explore the regenerating liver. These models have facilitated many discoveries, which have brought up new questions, new answers, and needs in experimental settings.

The mouse model has proven to be accessible and practical in these experimental settings. Mice are smaller in size and therefore, easier to handle especially in surgical procedures, cage management, postoperative visits, and interventions, such as injections and sample collection. One of these new needs, as mentioned previously, has been the need to examine the different genes and their regulations in the life course of the cells. Mouse models started to attract the attention of the researchers, because of their feasibility when using transgenic technologies. Being able to mute or overexpress specific genes in particular cells allows isolated observation of consequences from new therapeutic approaches or limiting factors of natural cell courses of tissues with impaired regenerative capacities.

By addressing the development and current state of the research of the now-popularized extended hepatectomy mouse model, this review summarizes the state of the art liver techniques and supports surgeons in avoiding potential pitfalls.

## Methods

### Boolean search algorithm

For the PubMed search conducted up to February 2019, the following Boolean search algorithm was used:

*((liver AND resection) OR hepatectomy) AND ((rodent AND model) OR (mice AND model) OR (mouse AND model) OR (murine AND model) OR mice OR mouse)*

#### Inclusion criteria

- The study is available in full text.
- The language of the study is either English or German.
- The animal model used in the study is a mouse model.
- The conducted surgery is a major hepatectomy, not exceeding 80%.

#### Exclusion criteria

- The study does not fulfil the inclusion criteria.
- The study does not reflect the current state of the art of the described animal model and is outdated by more recent publications with the same focus.
- The article is a systematic review.

In total, 21 of the shown filtered 3130 publications after the search have been identified as apposite to be included in this review and studied in full text. All legal requirements and the constitution of good clinical practice of the Charité – Universitätsmedizin Berlin were followed.

The photographs of the step-by-step instructions for extended liver resection in mice were taken as part of the training program (L0265/12) in the animal facilities of Charité—Universitätsmedizin Berlin. All procedures with the animals were approved by the Berlin State Office for Health and Social Affairs for Animal Welfare and Experimentation (LaGeSo Berlin).

## Results

As a result of the described PubMed search, the following articles in the table have been included in the review (Table 1). Different resection and anaesthesia methods, intervention durations, mortalities, animal choices, and resection extents with the postoperative observation time points have been listed and compared.

**Table 1:** Overview of the revised articles

Year	Work-group	Publication	Resection method	Duration of the intervention (min)	Anesthesia	Mouse			Extent of the liver resection (%)	Mortality	Observation time
						Strain	Age (w)	Weight (g)			
1953	Yokoyoma et al	Regeneration of mouse liver after partial hepatectomy	Suture ligature of left and medial lobe (Brues, Drury, and Brues)	–	Ether	Strain A white	13	24–27,5	65	–	3/4, 1, 2, 3, 4, 5, 6, 7, 8, 10, 14, 21, 28, 38–45 days 2, 4, 6 m
2004	Nikfarjam et al	A model of partial hepatectomy in mice	Hemostatic clip	–	Ketamine/ Xylazine + Carprofen	CBA	8–10	20–25	70	2%	21 days
2004	Garbow et al	MRI measurement of liver regeneration in mice following partial hepatectomy	Suture ligature of left lateral lobe Suture ligature of right median lobe Suture ligature of left median lobe	20–25	Isoflurane	–	–	–	67	–	–
2006	Inderbitzin et al	Regenerative capacity of individual liver lobes in the microsurgical mouse model	–	–	–	BALB/c	6–8	20–25	75	–	24, 48 h
2008	Mitchell et al	A reproducible and well–tolerated method for 2/3 partial hepatectomy in mice	Two Suture ligature	–	Isoflurane + Buprenorphine	–	8–14	–	67	–	–
2008	Boyce et al	A detailed methodology of partial hepatectomy in the mouse	Suture ligature	–	Isoflurane + Buprenorphine	–	–	–	70	4%	–
2008	Martins et al	Hepatic lobectomy and segmentectomy models using microsurgical techniques	Microscope assisted Catheter-guided Ligature	15	–	–	–	–	70	–	–
2009	Sorg et al	Consequences of surgical stress on the kinetics of skin wound healing: partial hepatectomy delays and functionally alters dermal repair	Suture ligature of right upper left upper and left lower lobe	–	Ketamine/Xylazine	SKH1-hr	8–12	35–45	70	0%	10 days
2010	Zhang et al	The benefits of ligating the lobar portal triads before partial hepatectomy in the mouse	Ligature of the lobar portal triads + Suture ligature of left lateral lobe and median lobes	15–20	Isoflurane + Bupivacaine	C57BL/6	8–12	20–25	70	1%	7d
2011	Hori et al	Simple and sure methodology for massive hepatectomy in the mouse	Suture ligature Hemostatic Clip Electrocautery scalpels	Suture: 30 Clip: 10	Isoflurane + Buprenorphine	C57BL/6	10–20	25	> 70	50%	180 h
2012	Bonninghof et al	Effect of different liver resection methods on liver damage and regeneration factors VEGF and FGF-2 in mice	Suture Ligature	–	Ketamine/ Xylazine + Buprenorphine	BALB/c	8–10	20–25	50 and 70	2%	10 min 3, 24, 48, 120, 240 h
2012	Hori et al	Simple and reproducible hepatectomy in the mouse using the clip technique	Suture ligature Hemostatic clip	Suture: 30 Clip: 10	Isoflurane + Buprenorphine	C57BL/6	10–20	25	75 and 80	75% resection: 40% 80% resection: 90%	180 h
2014	Xie et al	Monitoring of systemic and hepatic hemodynamic parameters in mice	–	–	–	–	–	–	–	–	–
2015	Nevzorova et al	Partial hepatectomy in mice	Suture ligature of left lateral lobe and left portion of the median lobe	–	Ketamine/xylazine and isoflurane	–	8–14			5%	0.5, 2, 4, 6 h
2016	Forbes et al	Liver regeneration—mechanisms and models to clinical application	–	–	–	–	–	–	–	–	–
2016	Orsini et al	MRI as Primary End Point for Pharmacologic Experiments of Liver Regeneration in a Murine Model of Partial Hepatectomy	Suture ligature of left lobe and median lobe	30	Isoflurane + Buprenorphine	C57BL/6 J	12–14	–		–	4 days
2016	Xu et al	A reliable scoring system after major liver resection in mice	Suture ligature of left lateral lobe and median lobe	–	Isoflurane + Buprenorphine	C57BL/6	10–12	19–23	65,30	24 h: 19% 2d: 28% 3d: 30% 4d: 33% 7d: 33%	6, 12, 24 h 2, 3, 5, 7 days
2016	Young et al	Metabolic scaling predicts post-hepatectomy liver regeneration after accounting for hepatocyte hypertrophy	–	–	–	–	8–12		68	–	0, 12, 24, 36, 48, 60, 72, 96 h
2016	Xie et al	Quantification of Hepatic Vascular and Parenchymal Regeneration in Mice	–	–	–	–	–	–	–	–	–
2017	Will et al	Longitudinal micro-computed tomography monitoring of progressive liver regeneration in a mouse model of partial hepatectomy	Suture ligature of upper left posterior and left anterior lobe	–	–	C57BL/6 N	11–12	18–26	42	–	1, 2, 3, 7 days
2018	Ozawa et al	Evaluation of safety for hepatectomy in a novel mouse model with non-alcoholic steatohepatitis	Single suture ligature of left and middle lobe	–	Ether	C57BL/6 J	6	–	70	0%	6, 12 h
							Ø 11	Ø 23			

A systematic search was conducted using Pubmed, gathering publications, which engaged with the mouse model of extended liver resection as a method itself. The results of this search were filtered according to their language, novelty, and relevancy. The Boolean search found 3741 articles on Pubmed, with 3130 publications remaining when filtered by language and the presence of a full text. In total, 21 of these publications examined the critical themes of the animal model described

One can see in Table 1 that the mortality of the listed publications varied from 0 to 50%, on the surgeon's experience and the method of resection. The liver resection was mainly performed with classic sutures (14 out of 21 publications). Hori et al. examined the mortality rates of the clip technique and the suture technique in 2 studies and could not show any difference (*p* < 0.05) [18, 19]. In total, the operating time for the clip technique was 10 min [18, 19] and for the classic suture technique 15–30 min [15, 18–22]. The anaesthesia was mainly carried out by inhalation with isoflurane (10 of 21) combined with analgesia. Ketamine/xylazine injections were mainly used during surgery (4 out of 21 publications) followed by post-surgical analgesia with buprenorphine (7 out of 21 publications) or metamizole. The most used mouse strain was C57BL/6 (7 of 21 publications) which was on average 11 weeks old with a weight of 23 g.

### Roots, new needs, and new establishments

Healthy, undamaged and fully functional liver in an adult tends to retain its volume in relation to the body size, in a serene state, where its cells are mitotically quiescent. Following any change in the architecture or damage, the liver attempts to recoup the lost cells by dividing. In order to investigate this phenomenon, Higgins and Anderson described and conducted the first liver resection model in rats in 1931, as a simulation of loss of the functional tissue [7]. Resection of the two anterior lobes of the existing four lobes would equate to a tissue reduction of 70% and provide the liver with the necessary inducement to proliferate and hypertrophy (Fig. 2). The regeneration peaks approximately 24 h after the resection, where the majority of the cells enter the S-phase and the DNA replicates [8]. It takes roughly eight days to reach 93% of the original liver size and 20 days to reach 100% of the original liver size [8]. According to the findings of the rat experiments, the process of the regenerating epithelial cells is conducted by the non-parenchymal cellular elements of the tissue: stellate cells, sinusoidal endothelial cells, and the macrophages. These discoveries lead researchers to ask how this complex process between organs and cellular elements is coupled and coordinated. Modification of the rat model with different constellations, such as Eck-fistula model, portal vein arterialization, portal vein ligation, auxiliary liver graft transplantation, and separation of the portal income from different gastrointestinal sections, revealed the role of factors, such as the amount of blood flowing through the liver after the tissue loss, the intra-portal pressure of this blood flow, the origin of the blood the liver receiving, whether it is arterial, portal or caval, and finally the oxygenation status and the substances, hormones, and nutrition supplied to the tissue with blood [9, 17]. In the light of these new findings and itemizations of humoral elements, new questions and new needs of experimental settings have arisen. To study the effects of cytokines, chemokines, hormones, and receptor molecules, genetic modification methods have been put forward by different workgroups. Due to the poorly developed technology in manipulating the rat genome, transgenic mice caught many researchers’ attention. The ability to overexpress or mute genes and change their products function or occurrence in the cells via transgenic strains enables to monitor regenerative processes under isolation, which leads to the establishment of the hepatectomy model in mice [8].

The surgical procedure in mice is almost the same as the procedure with rats, except for the anatomical differences and their consequences for the experimental setup. The two most significant anatomical differences between the mouse and rat, which make the operations not identical, are the absence of gallbladder in the rat abdomen and the number of main lobes. The rat liver has four main lobes, while the mouse liver is divided into five lobes [15, 16].

### State of the art

In the light of new necessities and demands, the liver resection model in mice has become the gold standard, by way of being easy to master and reproduce (Table 1). To engineer a successful and goal-driven experimental setting, one must discern the factors that influence the experimental conditions and control them. Essential factors for the experimental setting of the extended liver resection are gender, age, and the selected anaesthesia. It is useful to acknowledge that the female mice show a delayed and decreased replication and reconstruction rate, which could be argued with different sex hormones and in this way male mice are preferable over the female [23]. Besides involving mice of the same age, it is crucial to consider that the regeneration capacity and age are inversely proportional to each other and higher age is correlated with higher postoperative mortality [5].

The necessity of the anaesthesia has to be regarded and local law regulations of animal welfare have to be followed. Nonetheless, the choice of the anaesthesia should be made after consideration of the reduced hepatic clearance of the substance following the surgical procedure. The ideal anesthesia consists of maximal effect and minimal side effect of the favoured agents. A broad therapeutic index is desired, red and controllability should be easily managed via dose adjustment. The selected method of anaesthesia also needs to be reproducible and neutral on the parameters that are being measured. Nevertheless, no anaesthetic agent fulfils each of these criteria at the same time. This leads to the necessity of combining different agents for analgesia, hypnosis and muscle relaxation. Volatile anaesthetics with halogenated ether (such as Isoflurane and Sevoflurane) stand out in this context, with their tractability, adequate relaxation, and successful sedation effect on the mice. They have low metabolization, thus, are tolerable with other medicine and are easy to wake up from. However, the volatile agents do not provide the analgesia sufficiently. Therefore, they must be complemented by analgesic agents, which are mostly applied via injection (intravenous, intraperitoneal, intramuscular, or subcutaneous). A classic combination is xylazine and ketamine because of their applicability and controllability followed by post-surgical analgesia with buprenorphine or metamizole [5, 16].

One of the further points of decisions is the choice of surgical technique: Martins et al. described four different techniques, where some parts of the systematic overlap with the descriptions of other groups––classical suture technique, the hemostatic clip technique, the vessel-oriented parenchyma-preserving technique, and the vessel-oriented microsurgical technique [15]. The simplest one is the classical technique, where the liver mass is resected by a ligature. This method’s risks are the possible injuries and the vena cava stenosis, considering the liver tissue’s pedicular structure. A way of obviating these possible risks is modifying the technique with additional ligatures, instead of en-bloc resection. The hemostatic clip is to be favoured because of its time-saving aspects [18, 19], which brings different concerns about the possible immunological reactions due to an intra-abdominal foreign object [24]. These concerns, however, are not confirmed by further investigations. The vessel-oriented parenchyma-preserving technique appears to be an upgrade from the classical technique, with more appraisal of the liver tissue and vascular anatomy. The vessel-oriented microsurgical technique is supposed to be similar to the clinical liver resections, while ligating the portal vein and the hepatic artery branches prior to the tissue resection [15]. Finally, the extent of the liver resection can be varied considerably. However, a 70% extended hepatectomy proves to be optimal for the investigation of liver regeneration, while a 90% liver resection is associated with a very high mortality and is therefore unsuitable for the analysis of liver regeneration [18, 19].

#### Liver resection by classic suture technique:

**Fig. 1:**
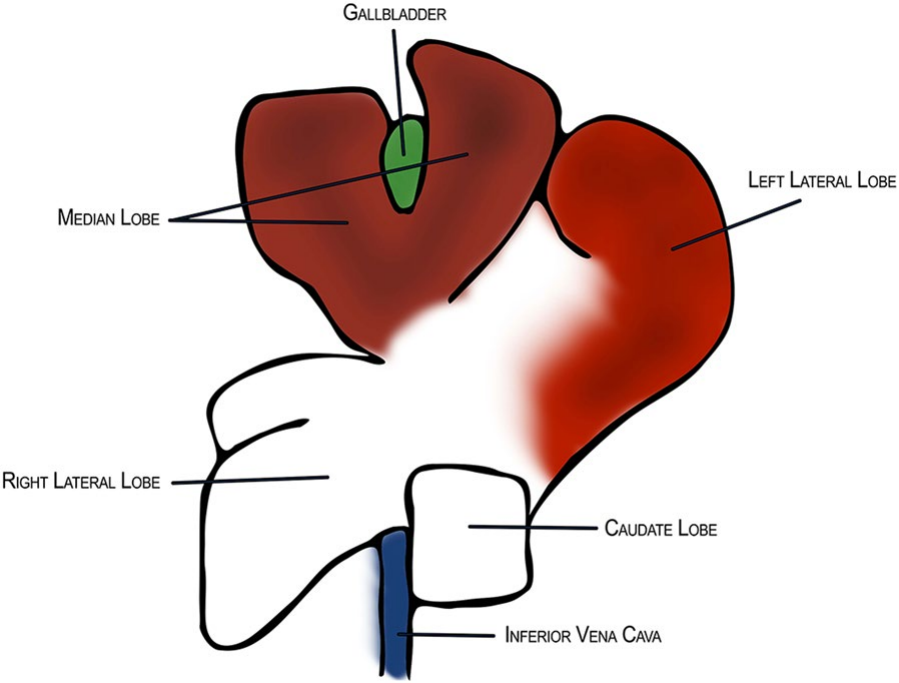
Schematic illustration of the lobes of the mouse liver

**Fig. 2:**
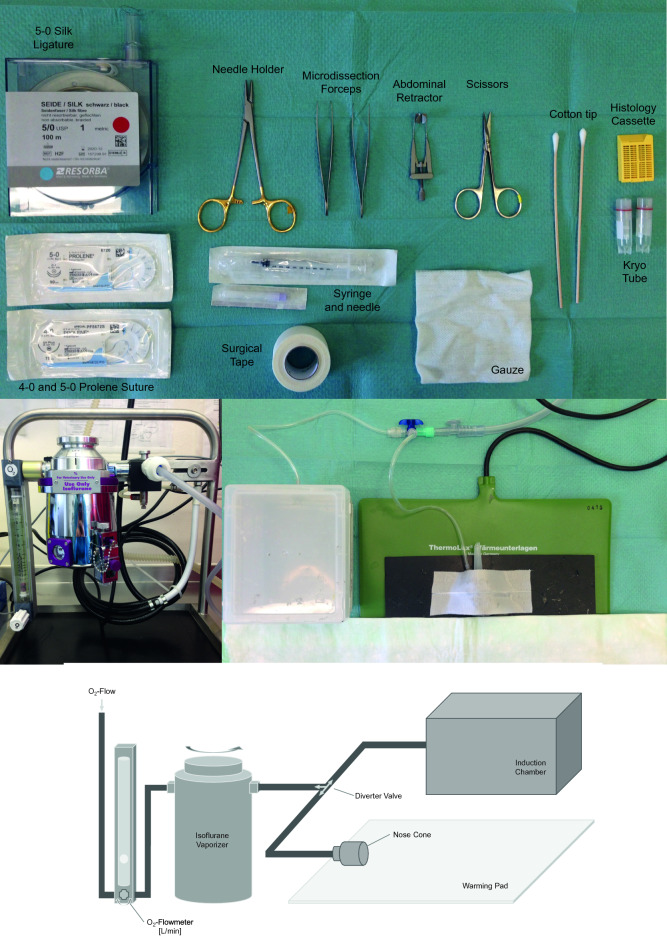
General setup for performing a liver resection, including the obligatory surgical instruments

**Fig. 3:**
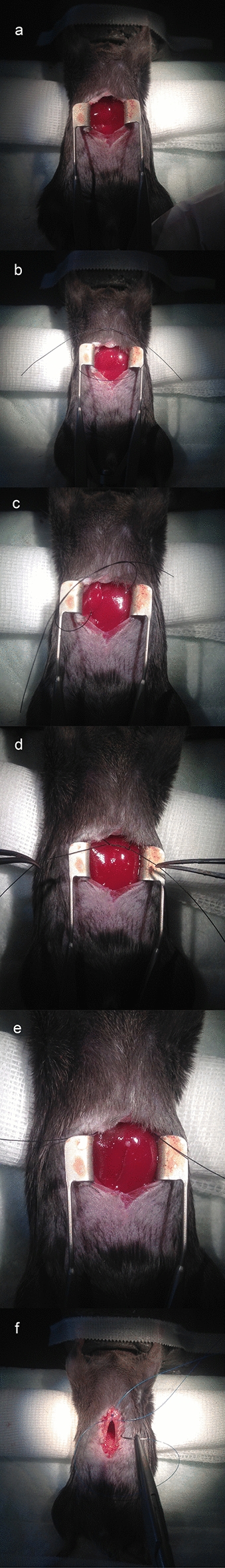
Surgical steps of an extended liver resection

Once the experimental setting, shown in Fig. 1, is prepared, the surgery begins with the induction of anaesthesia, which is described previously as a volatile isoflurane application (2% isoflurane, 2 L/min oxygen flow) and intraperitoneal application of ketamine/xylazine combination (100 mg/kg ketamine 5 mg/kg xylazine). Following the disinfection and the shave, midline abdominal incision (2.5 cm) reaching to the xiphoid process is made and the retractor of an adequate size is inserted to expose the liver (Fig. 3a), primarily the median lobe with the gallbladder and the left lateral lobe, illustrated in Fig. 2. Sequentially the liver tissue is inspected and the falciform ligament presented by gentle movements with a moistened cotton tip. The liver is mobilized from the falciform ligament and a pre-looped ligature (4–0 silk) (Fig. 3b) is gently inserted around the left lateral lobe as close as possible to the base with simultaneous gentle cotton-tip movements to clear the sight from the median lobe. Microdissection forceps are used to remove the ligated liver lobe. Next, another pre-looped ligature (4–0 silk) is set around the median lobe above the gallbladder with similar cotton-tip manoeuvre (Fig. 3c, d), but this time with additional attention not to obstruct the inferior vena cava (Fig. 3e). Resection cut is placed finally below the ligature and the tissue is scouted for complications. Optionally the abdomen can be washed and injected with glucose solution to prevent post-operative hypoglycemia. Ultimately, the peritoneum (Fig. 3f) and the skin are closed separately with 5–0 silk suture, postoperative analgesia injected subcutaneously in the neck, and the animal transported to the postoperative recovery area for further observation. The surgical procedure’s total duration is estimated as 15–20 min in the literature depending on the surgeon’s experience level. In order to serve the purpose of the surgical procedure, samples and data should be collected and evaluated systematically.

#### Postoperative care and experimental analysis

Non-invasive scoring systems [25], postoperative weight course, and survival curves are absolute necessities and other non-invasive imaging methods, such as magnetic resonance imaging [20, 22] and X-ray microtomography [26], are rather optional and cost-inefficient. CT has the radiation dose and MRI the additional volatile anaesthesia due to the prolonged imaging time as limiting factors. The most common way to collect samples is to sacrifice the animal. Contingent on hypothesis one may collect remnant liver tissue and blood, which are the most suitable for liver regeneration. Typical investigation time points are 6 h (priming phase), 36 h (onset of S-Phase), 48 h (peak of DNA replication), and 60 h (termination of cell cycle activity) [5]. The collected remnant liver tissue can be used for PCR and measurement of wet liver weight to body weight and dry liver weight to wet liver weight ratio, which gives information on the tissue growth, thus liver regeneration, cell proliferation, and hypertrophy. Histological and immunohistochemical findings with special staining methods, such as Ki67, PCNA, BrdU for cell proliferation, and Hematoxylin Eosin or Oil Red O for fatty degeneration, also serve to assess the regeneration [27]. Blood samples can be preserved and used for liver enzymes and inflammation parameters for liver function and systemic condition, furthermore for a screening of genes, cytokines growth factors, and more.

### Pitfalls

The extended liver resection in rodents, particularly in mice, is widely accepted as one of the best acute liver injury simulations and the subsequent liver regeneration. It allows researchers to inspect isolated genes and their products, manipulate the severity of the liver injury, and combine it with neoplastic alterations. Naturally, it comes with some pitfalls and limitations too. Although the non-invasive scoring systems are well described in the literature and obligated by law, in order to avoid animal suffering, the reproducibility and the objectivity of these scoring systems are still dependent on the conductor of the experiment.

As shown in Table 1, different research groups utilize two different techniques to resect the liver tissue (hemostatic clip vs suture). Hori et al. depict the contrast very well of these two methods via comparing the learning curves [18, 19]. Liver resection is eventually an advanced procedure that requires experience and a basic routine. In our institution the time of adaptation to perform the complex procedure of liver resection is well acknowledged, and thus, attendance to a training program under the supervision of an experienced surgeon is required before starting the main experiments.

The surgical procedure of extended liver resection in mice appears to be perceived as easy to learn and perform. However, avoiding the surgical complications, such as bleeding from the hepatic vessels caused by imprecise ligation of the liver lobes too close the vein star, obliteration of the inferior vena cava, cholestasis following the removal of the gallbladder with the median lobe, undesirable postoperative acute liver failure due to the inadequate remnant liver volume, impaired wound healing, and abdominal wall dehiscence of weak sutures, which seem to be the main concerns of almost all of the publications and new approaches to this method [5, 18, 19, 28–30].

Most severe complications occur when the sutures are unduly tight and cause bleeding from the liver parenchyma and blood vessels with sheer stress. This method’s modifications with sewing the lobar portal triads before the actual hepatic resection or microsurgery provide better sight and manoeuvring [7, 9]. Individualizing the dissections with ligature of vascular and biliary branches reduces the amount of devascularized liver, cholestatic complications, inflammation with or without infection, and vena cava constriction [7]. In order to avoid abdominal wall dehiscence, separate sutures (5–0) of peritoneum and skin with additional skin adhesive are described as safety precautions.

Depending on the number of groups, time points, and the number of animals in one group, the in toto operation becomes time consuming. One may choose to operate the mice of the same time point and different groups, at the same time in order to minimize the non-conformance between each operated mouse. This has the advantage of a structured operation, reduction of surgeons influence, and more systematic approach in the postoperative course, where measurements are done concurrently, albeit the disadvantage of loss of concentration toward the end, which might, according to personal experiences, lower the quality of the individual operative outcomes. Another practical challenge is to optimize the cage size and number of animals in a cage. In order to follow the nutrition uptake precisely, cage size could be reduced to one animal per cage, which may not always be feasible, put the animals under social stress in consequence of the iatrogenic isolation, and affect their recovery process.

The three Rs (Replacement, Reduction, and Refinement) principles in the animal experiments have been developed 50 years ago and aim to provide a framework of more humane ethical and better animal experiments [31]. These principles were first presented and elaborated in “The Principles of Humane Experimental Technique” by Russel and Burch [32]. In this context the number of animals in the experimental settings is kept as low as possible and as much as needed. One may face difficulties to make clear statements based on the results due to the small number of cases, which makes it challenging to distinguish between whether the findings are mere coincidence or show real significance. Furthermore, the translation of the results obtained by the animal experiments should be done carefully. Biochemical and physiological interactions on the way to the liver regeneration show differences between humans and rodents. The critical future liver remnant volumes lie around 90–95% in rodents and 70–80% in humans. Thus, the conducted extended liver surgery in mice would be most likely fatal for humans [30].

## Discussion

Mouse models of liver resection appear to be in full vogue since the 1950s among the circles researching regenerative potentials, injuries, and surgeries of liver, on the grounds that they offer a great versatility in genetic manipulation and relatively easy learning curves with fast and reliable results. The conducted PubMed research yields an overview of extended liver surgery in mice. In total, 21 publications were identified from a Boolean search on Pubmed which engaged with the mouse model of extended liver resection as a method itself. Through the overview, this paper reviews the shift of the extended liver resection model from rat to the mouse, describes the state of the art in the experimental setting, and discusses the possible limitations and pitfalls. Clearly, the extended liver resection in mice is a reproducible, practical and easy to learn method.

The mortality varied from 0 to 50% depending on the surgeon's experience, the method, and liver volume of the resection (Table 1). Unfortunately, the studies’ reasons for this enormous variance cannot be explained and reflect a general problem in experimental surgery: The robustness and reproducibility of results when applying the same experimental method. In fact, complication management, failure to rescue, is well described in human medicine, but is often not mentioned in any laboratory experiments [33]. One of the reasons for survival difference could be the learning curve, which shows a sharp rise, especially at the beginning of learning. In fact, it is often the younger colleagues in the laboratory who have to perform the liver resection. Hori et al. inspected the learning curves and showed significantly different survival curves after beginning to learn the liver resection compared to more experienced learning levels either with the suture or clip technique (*p* < 0.05) [18, 19]. While the survival rate after an 80% liver resection reached a plateau after 30 cases with the suture technique, only 11 cases were required with the clip technique to reach a steady state. Although the same survival rate can be achieved with both techniques, the operative time was significantly shorter by the clip technique [18, 19]. Another reason for the high variance in mortality is possibly the surgical accuracy or a different definition of the liver’s percentage that was removed (Table 1).

The liver resection was mainly performed with classic sutures (14 out of 21 publications). The reason for this is certainly due to the practicality and cost-effectiveness of the ligatures compared to the use of clips. However, most of the reviewed articles do not clarify the reasoning behind their decision of the surgical technique. The matter of clips impairing the liver regeneration as foreign objects in the abdominal cavity and inducing immune reactions has not been supported with any evidence. One group suggests the possibility of clips being dislocated by aggressive movements once the mouse recovers from the surgery and anesthesia, which evokes the consideration of different clip sizes [13]. As opposed to the hemostatic clips, suture ligations require adroitness in handling the delicate material. This might be interpreted as time consuming, which again can be managed with exercise.

Isoflurane was used mainly for anaesthesia (10 out of 21 publications) combined with buprenorphine or ketamine/xylazine. The selection of drug regimes was likely based on experience and knowledge gained from human studies. A comparison between certain drugs was not presented in the studies examined. Moreover, monitoring the vital parameters and the depth of the anesthesia plays a vital role and a challenge especially when one person conducts the procedure. The most commonly used mouse strain was C57BL/6 (7 of 21 publications), indicating that this line is robust for major surgery. The choice of the mouse strain, however, must be clearly adapted to the research question.

Some findings point toward a link between portal pressure applied by specific lobes during the resection and possible shear stress on the vessels. This might influence the regenerative response of the liver due to lobe-specific regeneration. This indication casts doubts on the concept of homogenous liver regeneration [4]. In this manner, the choice of the lobe that is going to be resected and the method of tissue separation gain importance.

An important aspect that provides an ethical framework to the animal experiments and should always be kept in mind is the principle of three Rs [32]. One should replace the animal experiments as far as they can be represented with other methods, e.g., mathematical models and cell cultures. If animals’ use is irreplaceable, the number of animals should be reduced, which could eventually jeopardize the strength of the statistical assertion and become one of the pitfalls. Lastly animal safety and wellbeing should be prioritized and documented cautiously, as in refine.

## Conclusion

In this systematic review the wide range of possibilities in experimental models of liver resection with mice has been depicted to obtain a good overview. With the new technologies in genetic engineering and surgical instruments, the surgeon found many ways to personalize an experimental setting. Additionally, the surgeon realized the role of various systemic or cell-specific knockouts could be validated for liver regeneration. Extended liver resection in mice provides a reproducible, practical and easy-to-learn method.

## Data Availability

Not applicable.
